# CK2 Inhibitor CX-4945 Blocks TGF-β1-Induced Epithelial-to-Mesenchymal Transition in A549 Human Lung Adenocarcinoma Cells

**DOI:** 10.1371/journal.pone.0074342

**Published:** 2013-09-04

**Authors:** Jiyeon Kim, Seong Hwan Kim

**Affiliations:** Laboratory of Translational Therapeutics, Pharmacology Research Center, Division of Drug Discovery Research, Korea Research Institute of Chemical Technology, Yuseong-gu, Daejeon, Republic of Korea; National Cancer Center, Japan

## Abstract

**Background:**

The epithelial-to-mesenchymal transition (EMT) is a major phenotype of cancer metastasis and invasion. As a druggable cancer target, the inhibition of protein kinase CK2 (formally named to casein kinase 2) has been suggested as a promising therapeutic strategy to treat EMT-controlled cancer metastasis. This study aimed to evaluate the effect of the CK2 inhibitor CX-4945 on the processes of cancer migration and invasion during the EMT in A549 human lung adenocarcinoma cells.

**Materials and Methods:**

The effect of CX-4945 on TGF-β1-induced EMT was evaluated in A549 cells treated with TGF-β1 (5 ng/ml) and CX-4945. The effect of CX-4945 on TGF-β1-induced cadherin switch and activation of key signaling molecules involved in Smad, non-Smad, Wnt and focal adhesion signaling pathways were investigated by Western blot analysis, immunocytochemistry and reporter assay. Additionally, the effect of CX-4945 on TGF-β1-induced migration and invasion was investigated by wound healing assay, Boyden chamber assay, gelatin zymography, and the quantitative real-time PCR.

**Results:**

CX-4945 inhibits the TGF-β1-induced cadherin switch and the activation of key signaling molecules involved in Smad (Smad2/3, Twist and Snail), non-Smad (Akt and Erk), Wnt (β-catenin) and focal adhesion signaling pathways (FAK, Src and paxillin) that cooperatively regulate the overall process of EMT. As a result, CX-4945 inhibits the migration and invasion of A549 cells accompanied with the downregulation of MMP-2 and 9.

**Conclusions:**

Clinical evaluation of CX-4945 in humans as a single agent in solid tumors and multiple myeloma has established its promising pharmacokinetic, pharmacodynamic, and safety profiles. Beyond regression of tumor mass, CX-4945 may be advanced as a new therapy for cancer metastasis and EMT-related disorders.

## Introduction

The epithelial-to-mesenchymal transition (EMT) is a major phenotype of cancer metastasis and invasion that occurs in epithelial tumors and accounts for 90% of human tumors [1–4]. EMT is characterized by the loss of epithelial characteristics and the acquisition of mesenchymal characteristics; loss of epithelial markers such as E-cadherin and the induction of mesenchymal markers including N-cadherin and vimentin are hallmark early- and late-stage events of EMT, respectively. Morphologically, cancer cells change from a polarized, epithelial shape to a spindle-shaped phenotype. Epithelial tumor cells become more motile and invasive after undergoing EMT [5–7].

Various growth and differentiation factors can induce or regulate the process of EMT in cancers [8,9]. Tumor growth factor (TGF)-β has received much attention as a characterized inducer of EMT during cancer progression and metastasis [9]. TGF-β triggers the signal for EMT through a heteromeric complex of two type I and two type II transmembrane serine/threonine kinase receptors. TGF-β-induced activation of the receptor complex leads to the activation of Smad2 and Smad3 through phosphorylation of the type I receptors. Next, trimers consisting of phosphorylated Smad2/3 and Smad4 translocate to the nucleus, where they cooperate with transcription factors such as Snail and Twist to repress the expression of epithelial markers and activate the expression of mesenchymal markers at the mRNA level [10–12]. This signaling is referred to as TGF-β-activated Smad signaling in EMT.

In addition to activating the Smad2/3-dependent pathway, TGF-β can also activate non-Smad signaling pathways that are activated by tyrosine kinase receptors or other receptor types in response to their respective ligands, which are classified outside the TGF-β family [13–16]. For example, TGF-β-induced activation of Akt and ERK pathways has been linked to the characteristics of EMT, such as cytoskeletal organization and cell growth, survival, migration, and invasion [17]. Non-Smad signaling pathways cooperate with TGF-β/Smad signaling to constitute TGF-β-induced EMT.

Wnt signaling can also cooperate with TGF-β signaling during elaboration of the EMT response. Although secreted Wnt proteins do not induce EMT, their canonical signal controller, β-catenin, links E-cadherin to the cytoskeleton and functions as a component of cell-cell adhesion junctions to undertake the epithelial phenotype of adherence. However, in response to TGF-β, the nuclear localization of β-catenin induces the transcription of genes required for tumor migration and invasion [18].

Tumor migration and invasion by TGF-β-induced crosstalk between signaling pathways, including Smad, non-Smad and Wnt signaling pathways, accompany the increased expression and activity of matrix metalloproteinases (MMPs), which have been recognized as major contributors to the proteolytic degradation of the extracellular matrix that is required for tumor cell migration and invasion [19]. Additionally, focal adhesion kinase (FAK), Src, and paxillin are functionally interdependent molecules related to EMT-mediated tumor cell migration and invasion [20].

As mentioned above, the processes of EMT-mediated tumor cell migration and invasion are regulated in a complex manner by several molecules and signals. To control both tumor metastasis and tumor growth, the upstream signaling molecules involved in this process (e.g. protein kinase CK2) have been considered potentially druggable target molecules. CK2, a serine/threonine kinase, plays a pivotal role in many cellular events, including cell cycle, differentiation, and proliferation, by regulating the crosstalk between multiple signaling pathways (e.g. PI3K/Akt, Wnt, and NF-κB) [21–23]. Structurally, CK2 is consists of two catalytic subunits (α and α´) and two regulatory subunits (β and β´). The two catalytic subunits are linked to each other through the β subunits, and this linkage acts a key locus for CK2-mediated signaling in the nucleus [24,25]. A recent study has reported that CK2α modulates cell proliferation and invasion by regulating EMT-related genes [26]. Additionally, an imbalance of CK2 subunits resulting in the decrease of CK2β has been correlated with the induction of EMT-related markers, and CK2β-depleted epithelial cells display Snail-dependent EMT characteristics (e.g. morphological changes, enhanced migration, and anchorage-independent growth) [27]. These results suggest that the inhibition of CK2 may be a promising therapeutic strategy for treating EMT-related disorders including cancer metastasis.

**Figure 1:**
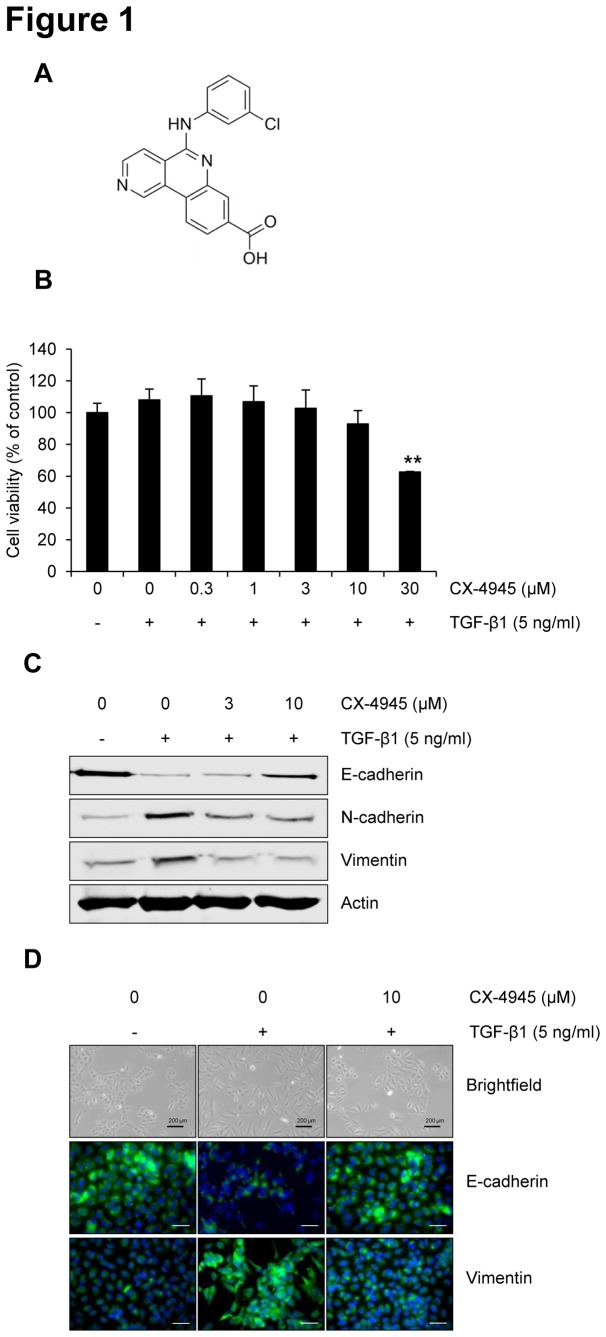
CX-4945 inhibits TGF-β1-induced EMT. (A) The chemical structure of CX-4945. (B) The effect of CX-4945 on the viability of A549 cells in the presence of TGF-β1. Briefly, cells wre treated with TGF-β1 (5 ng/ml) alone or in combination with CX-4945 for 72 h, and then cell viability was measured. **, *p* < 0.01 (versus the control). The effect of CX-4945 on the TGF-β1-induced expression of epithelial and mesenchymal markers was evaluated by Western blot analysis (C) and immunocytochemistry (D). Briefly, after 24 h serum starvation, A549 cells were treated with TGF-β1 (5 ng/ml) alone or in combination with CX-4945 in media containing 0.1% FBS for 72 h. All scale bars represent 200 µm.

Recently, we reviewed the druggability of CX-4945 (Figure 1A), a potent and selective ATP-competitive inhibitor of CK2 developed by Cylene Pharmaceuticals for testing in human clinical trials as an anti-cancer drug [28]. However, no promising data has been reported to demonstrate the potential for CX-4945 to inhibit the process of EMT. This study is the first to report that CX-4945 blocks the TGF-β1-induced EMT in A549 human lung adenocarcinoma cells by regulating Smad, non-Smad, Wnt, and focal adhesion signaling pathways.

## Materials and Methods

### Cell culture

A549 human lung epithelial adenocarcinoma cells were maintained in Dulbecco’s modified Eagle’s medium (DMEM; Hyclone, UT, USA) containing 10% heat-inactivated fetal bovine serum (FBS) and 1% antibiotics (100 U/ml penicillin and 100 µg/ml streptomycin) in a humidified atmosphere of 5% CO_2_ at 37^°^C.

### Cell viability assay

A549 cells (5 × 10^3^ cells/well) were seeded in a 96-well plate and incubated for 24 h. After serum starvation for 24 h, cells were treated with 5 ng/ml human recombinant TGF-β1 (R&D Systems, MN, USA) and CX-4945 for 48 h. CX-4945 was purchased from Sequoia Research Products (Pangbourne, UK). Cell viability was measured using a Cell Counting Kit-8 (Dojindo Molecular Technologies, ML, USA) according to the manufacturer’s instructions. The absorbance was measured using a Wallac EnVision microplate reader (PerkinElmer, Finland). All experiments were performed in triplicate.

### Western blot analysis

Cytoplasmic or nuclear protein fractions of A549 cells were prepared using a NucBuster Protein Extraction kit (Novagen, Germany). After protein quantification, cytoplasmic or nuclear proteins (40 µg) were loaded onto 10% polyacrylamide gels, and separated proteins were transferred to PVDF membranes. Expression of each protein was detected using a primary antibody. Antibodies raised against phosphorylated (p)-Akt (S129, T308 or S473), Akt, p-p21, p21, p-ERK1/2, ERK, p-Smad (S465/467), Smad2/3, Snail, p-FAK (Y925), FAK, p-Src (Y416), Src, p-paxillin (Y118), and paxillin were purchased from Cell Signaling Technology, Inc. (MA, USA). Antibodies raised against vimentin, β-catenin, Twist, and actin were purchased from Santa Cruz Biotechnology, Inc. (TX, USA). An antibody raised against E-cadherin was purchased from BD Transduction and antibodies raised against N-cadherin, CK2α (cat. No. 218703) and CK2β (cat. No. 218712) were purchased from Millipore (MA, USA), respectively. After incubation with HRP-conjugated secondary antibodies, membranes were developed using SuperSignal West Femto Maximum Sensitivity Substrate (Pierce, USA) and the LAS-3000 luminescent image analyzer (Fuji Photo Film Co., Ltd., Japan). ImageJ software-based quantification of the detected band was carried out and the relative, normalized ratio between phosphorylated protein and the protein itself or actin was presented in the figures.

### Immunocytochemistry

A549 cells (5 × 10^4^ cells) were incubated on a 1% gelatin-coated slide glass for 24 h. After serum starvation for 24 h, cells were treated with TGF-β1 (5 ng/ml) and CX-4945 for 48 h. All cells were fixed with 4% paraformaldehyde, permeabilized with 0.1% Triton X-100 in PBS, and blocked with 3% BSA in PBS. Expression of E-cadherin, vimentin, Smad2/3 and β-catenin was detected using each respective primary antibody and visualized with Alexa Fluor 488-conjugated secondary antibodies (Invitrogen, CA, USA). Nuclei were counterstained with Hoechst 33258. All scale bars represent 200 µm. All images were observed by DeltaVision RT wide-field epifluorescence microscope imaging system and softWoRxs image analysis program (Applied Precision, NW, USA).

### Migration assay

A549 cells (1.5 × 10^4^ cells/well) were incubated for 24 h in a bottom line-marked 96-well ImageLock Microplate (ESSEN BioScience, Inc., USA). After 24 h of serum starvation, wounds were made using a 96-well WoundMaker^TM^ (ESSEN BioScience, Inc., USA) and cells were treated with TGF-β1 (5 ng/ml) and CX-4945. The continuous kinetic output of migrated cells was analyzed every 6 h using IncuCyte^TM^ software. The relative wound density (RWD) was calculated in comparison with a similar cell population treated with TGF-β1 alone. All experiments were performed in triplicate.

### Invasion assay

A549 cells (5 × 10^4^ cells/well) were incubated in a 24-well plate for 24 h. After serum starvation for 24 h, cells were treated with TGF-β1 (5 ng/ml) and CX-4945 for 48 h. Cells were collected using trypsin-EDTA and resuspended in serum-free medium for counting. Next, 30 µl of culture medium containing 10% FBS was added to the bottom of Boyden chamber. After placing the gelatin-coated membrane filter, the silicone gasket, and the top chamber, the cell suspension (1 × 10^4^ cells/50 µl) was added to the top chamber, followed by incubation at 37^°^C in a 5% CO_2_ atmosphere for 6 h. The membrane filter was collected, fixed, and stained using the Diff-Quick staining kit (Dade Behring, NJ) according to the manufacturer’s instructions. After the filter was dried and stabilized on a glass slide using 30% glycerol solution, the migrated cells were counted in three randomly selected fields at 400× magnification. All experiments were performed in triplicate.

### Gelatin zymography

To analyze MMP-2 and MMP-9 activity in A549 cells, we incubated A549 cells (1 × 10^5^ cells/well) in a 24-well plate for 24 h. After serum starvation for 24 h, cells were treated with TGF-β1 (5 ng/ml) and CX-4945 in media containing 0.1% FBS for 48 h. The supernatants were collected, centrifuged at 3,000 × *g* for 10 min, concentrated using Amicon Ultra Centrifugal Filter Units (Millipore, Billerica, MA, USA), and quantified using the BCA protein assay kit (Pierce, USA). Proteins (40 µg) were loaded onto a gelatin-containing gel (8% acrylamide gel containing 1.5 mg/ml gelatin) and separated by electrophoresis. Next, the gel was washed with 2.5% Tween-20 solution, developed overnight at 37^°^C in Zymogram incubation buffer (50 mM Tris-HCl, pH 7.6; and 5 mM CaCl_2_), stained with 0.25% Coomassie blue R250 solution, and destained with a solution of 50% methanol and 10% acetic acid until the part of membrane degraded by MMP-2 or MMP-9 became clear.

### Quantitative Real-time PCR analysis

A549 cells (3 × 10^5^ cells/well) were incubated in a 6-well plate for 24 h. After 24 h of serum starvation, cells were treated with TGF-β1 (5 ng/ml) and CX-4945 in media containing 0.1% serum for 48 h. Total RNA was isolated using TRIzol reagent (Life Technologies, MD, USA), and cDNA was synthesized using the Omniscript Reverse Transcriptase Kit (Qiagen, CA, USA) according to the manufacturer’s instructions. Quantitative PCR was performed using Brilliant SYBR Green Master Mix (Stratagene, CA, USA) and the Mx3000P Real-Time PCR system (Stratagene, CA, USA) according to the manufacturer’s protocols. Primer sequences used in this study were designed as follows: MMP-2 forward, 5’-TTG ACG GTA AGG ACG GAC TC-3’; MMP-2 reverse, 5’-ACT TGC AGT ACT CCC CAT CG-3’; MMP-9 forward, 5’- TTG ACA GCG ACA AGA AGT GG-3’; MMP-9 reverse, 5’-GCC ATT CAC GTC GTC CTT AT-3’; Glyceraldehyde-3-phosphate dehydrogenase (GAPDH) forward, 5’-GAG TCA ACG GAT TTG GTC GT-3’; GAPDH reverse, 5’-GAT CTC GCT CCT GGA AGA TG-3’. All reactions were run in triplicate, and data were analyzed using the 2^−ΔΔC^_T_ method [29]. GAPDH was used as an internal standard. Statistical significance was determined using Student’s *t*-test with GAPDH-normalized 2^−ΔΔC^_T_ values.

### β-catenin reporter luciferase assay

A549 cells were transfected using SureENTRY transduction reagent with Cignal Lenti β-catenin reporter (5 × 10^5^ TU; SABioscience, MD) in an antibiotic-free medium. After 48 h, the medium was changed to culture medium containing 10% FBS, and transduced cells were selected in culture medium with 30 µg/ml of puromycin (Sigma, MO). After puromycin selection, cells (1 × 10^4^ cells/well) were incubated in a 96-well plate for 24 h. After serum starvation for 24 h, cells were treated with TGF-β1 and CX-4945 for 72 h. Then, β-catenin luciferase activity was evaluated using the luciferase reporter assay (Promega, WI). All experiments were performed in triplicate.

### Human phospho-kinase array

The levels of phosphorylated kinases resulting from stimulation with TGF-β1 were detected using the Proteome Profiler^TM^ Antibody Array Kit (R&D Systems, USA). A549 cells were incubated in a 6-well plate in medium containing 10% FBS for 24 h. Next, cells were replenished with serum-free medium for 24 h and treated with TGF-β1 (2 ng/ml) for 48 h. After incubation, all experimental procedures were completed according to the manufacturer’s instructions.

### Statistical analysis

Data are presented as mean ± SD. Statistical significance was determined using Student’s *t*-test, and differences were considered significant when ^##^, *p* < 0.01; ^###^, *p* < 0.001 (versus the control); *, *p* < 0.05; **, *p* < 0.01; ***, *p* < 0.001 (versus the cell population treated with TGF-β1 alone).

## Results

### CX-4945 inhibits the TGF-β1-induced cadherin switch

Before assessing the effect of CX-4945 on TGF-β1-induced EMT in A549 cells, we performed a cell viability assay to determine the concentration of CX-4945 to use in this study. Because there were no significant differences on cell viability at concentrations up to 10 µM in the presence of TGF-β1 (Figure 1B), we used up to 10 µM CX-4945 in the following experiments.

The effect of CX-4945 on TGF-β1-induced EMT was first evaluated by checking the cadherin switch (Figure 1C). In response to TGF-β1, E-cadherin was decreased, but N-cadherin was induced. However, 10 µM CX-4945 inhibited the TGF-β1-induced cadherin switch. Another mesenchymal marker, vimentin, was also induced by TGF-β1; however, its induction was inhibited by CX-4945. The inhibitory effect of CX-4945 on TGF-β1-induced EMT was also confirmed by visualizing the expression of markers (Figure 1D); consistent with Figure 1C, CX-4945 inhibited the decrease of E-cadherin and increase of vimentin that were induced by TGF-β1. We also observed that CX-4945 attenuated TGF-β1-induced morphological changes from cuboidal epithelial cells to fibroblast-like spindle-shaped cells.

### CX-4945 inhibits TGF-β1-induced migration and invasion through FAK/Src-paxillin signaling

**Figure 2:**
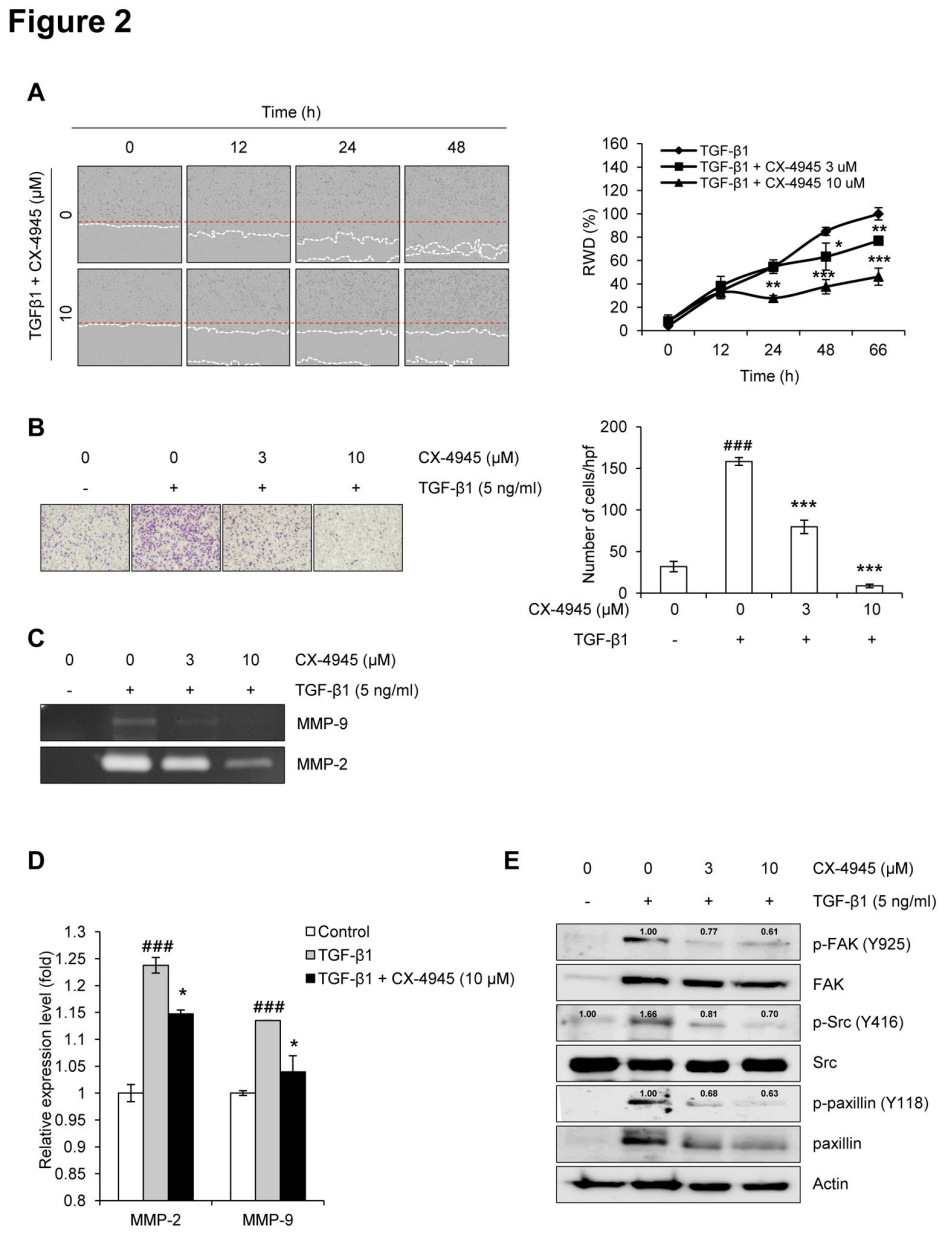
CX-4945 inhibits TGF-β1-induced migration and invasion. The effect of CX-4945 on the TGF-β1-induced migration and invasion of A549 cells was evaluated using IncuCyte software (A) and Boyden chambers (B), respectively. The red and white dashed line (A) represent the wounded area and the edge of migrated cells, respectively. Values (% RWD; Relative Wound Density) represent mean ± SD of triplicate samples and reported images are representatives of triplicate experiments. The effect of CX-4945 on the TGF-β1-induced activation of MMP-2/9 was evaluated using gelatin zymography (C), while its effect on TGF-β1-induced MMP-2/9 transcription was evaluated using real-time PCR (D). The effect of CX-4945 on the TGF-β1-induced activation of molecules such as FAK, Src, and paxillin was evaluated using Western blot analysis (E). Briefly, proteins were prepared in serum-deprived A549 cells treated with TGF-β1 (5 ng/ml) alone or in combination with CX-4945 in serum-free media for 48 h. Actin was used as a loading control. The relative, normalized ratio between phosphorylated protein and the protein itself was presented.

Next, we examined the effect of CX-4945 on the TGF-β1-induced migration and invasion of A549 cells. The wound healing assay revealed that CX-4945 inhibited the TGF-β1-induced migration of A549 cells (Figure 2A). In the Boyden chamber assay, CX4945 significantly inhibited the TGF-β1-induced invasion of A549 cells across the gelatin coated-membrane (Figure 2B). In addition, we examined whether CX-4945 inhibits the activation of gelatinases such as MMP-2 and MMP-9 and the expression of their mRNAs. TGF-β1 strongly induced the activation of MMP-2 and MMP-9; however, CX-4945 dramatically inhibited the induction of those factors (Figure 2C). CX-4945 also significantly inhibited the TGF-β1-induced expression of both MMP-2 and MMP-9 mRNAs. These results demonstrate that CX-4945 has the potential to inhibit TGF-β1-induced EMT in A549 cells without any cytotoxicity at concentrations of up to 10 µM.

The inhibitory effect of CX-4945 on the TGF-β1-induced invasion of A549 cells was further confirmed by evaluating the activation of the cancer invasion-related molecules FAK, Src, and paxillin [30]. Although TGF-β1 strongly induced the expression and phosphorylation of FAK and paxillin, those inductions were inhibited by the addition of CX-4945 (Figure 2E). Src expression was unchanged by exposure to TGF-β1 or TGF-β1 plus CX-4945. However, the phosphorylation of Src was induced by TGF-β1, and this induction was also inhibited by the addition of CX-4945. Similarly with these results, the TGF-β1-induced phosphorylation of FAK and Src, and expression of α-smooth muscle action (SMA) were strongly inhibited in human normal lung fibroblast CCD-18Lu by CX-4945 (Figure S1). Alpha-SMA is a biomarker in the process of TGF-β1-induced EMT of normal cells (also called to ‘fibrosis’). Additionally, the phosphorylation and expression of paxillin was not induced by TGF-β1, but those were also strongly inhibited by CX-4945. These results suggest that the expression and/or activation of FAK, Src, and paxillin are involved in the inhibitory effect of CX-4945 on the TGF-β1-induced invasion of A549 cells, and also suggest that its inhibitory effect of TGF-β1-induced EMT could be not cell line-dependent.

Since NF-κB has been well-known transcription factor involved in the process of tumor metastasis, the effect of CX-4945 on the transcriptional activation of NF-κB was additionally evaluated by the luciferase assay. TGF-β1 strongly induced the transcriptional activation of NF-κB, but CX-4945 significantly inhibited that at the low concentration (Figure S2). This result suggested the partial involvement of NF-κB in the anti-EMT activity of CX-4945, especially, its anti-invasion/migration action.

### CX-4945 inhibits TGF-β1-induced activation of Smad signaling

**Figure 3:**
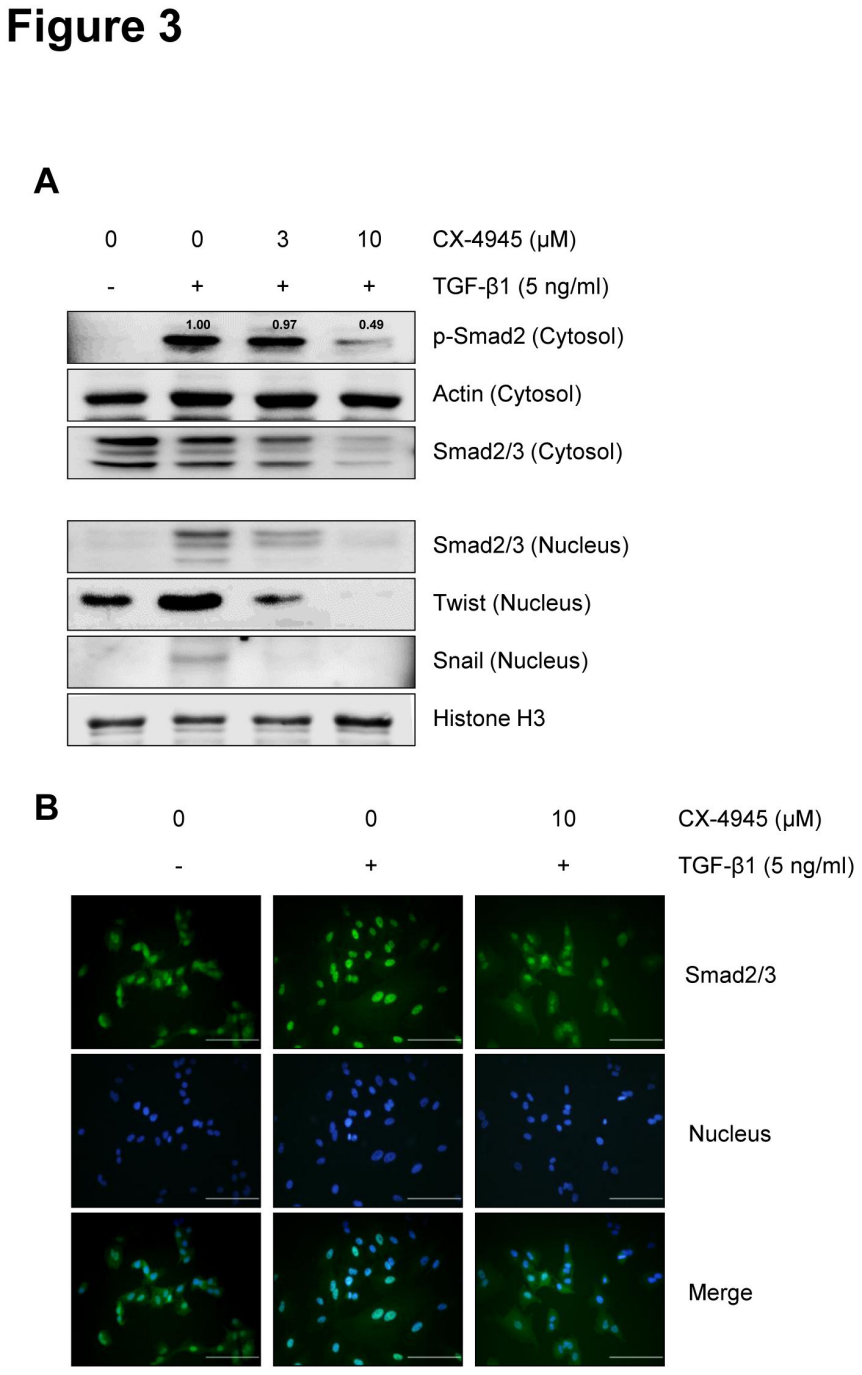
CX-4945 inhibits TGF-β1-induced Smad signaling. (A) The effect of CX-4945 on TGF-β1-induced activation of Smad and expression of Snail and Twist was evaluated using Western blot analysis. Briefly, after 24 h serum starvation, A549 cells were treated with TGF-β1 (5 ng/ml) alone or in combination with CX-4945 in media containing 0.1% FBS for 48 h. Cytosolic and Nuclear fractions were obtained as described in ‘Materials and Methods’. Actin and histone H3 were used as internal control of cytosolic and nuclear fraction, respectively. The relative, normalized ratio between p-Smad2 and actin was presented. (B) Nuclear translocation of Smad2/3 and its inhibition by CX-4945 were confirmed by immunocytochemistry. Nuclei were counterstained with Hoechst 33342. All scale bars represent 50 µm.

To elucidate the anti-EMT mode of action of CX-4945 in detail, we first tested the effect of CX-4945 on TGF-β1-induced activation of Smad signaling. In the TGF-β-mediated EMT processes, the phosphorylated Smad2/3/4 complex activates the transcription of EMT-related genes through interactions with other DNA-binding transcription factors such as Snail and Twist [31–34]. Here, TGF-β1 induced the phosphorylation of Smad2 and the nuclear translocation of cytosolic Smad2/3, and CX-4945 inhibited those inductions in a dose-dependent manner (Figure 3A). Additionally, CX-4945 inhibited the TGF-β1-mediated induction of nuclear Snail and Twist. The inhibitory effect of CX-4945 on the TGF-β1-induced nuclear translocation of cytosolic Smad2/3 was also confirmed using immunofluorescence microscopy (Figure 3B). Taken together, these results suggest that CX-4945 might inhibit TGF-β1-induced EMT by blocking the TGF-β1-induced canonical Smad signaling in A549 cells.

### CX-4945 inhibits TGF-β1-induced activation of the non-Smad Akt and ERK signaling pathways

**Figure 4:**
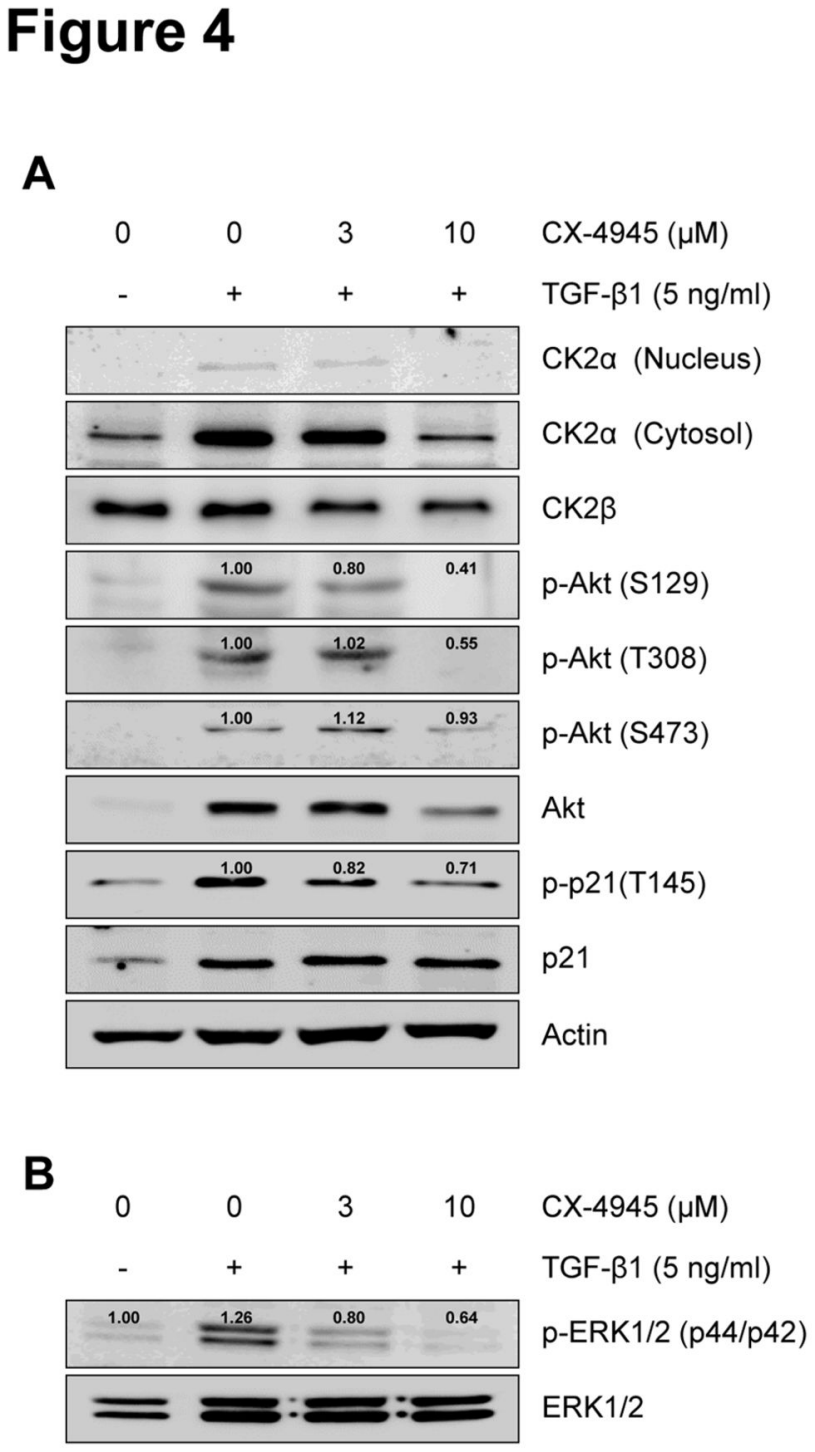
CX-4945 inhibits TGF-β1-induced non-Smad signaling. The effect of CX-4945 on TGF-β1-induced non-Smad activation was evaluated. Briefly, after 24 h serum starvation, A549 cells were treated with TGF-β1 (5 ng/ml) alone or in combination with CX-4945 in media containing 0.1% FBS for 72 h (A) or 48 h (B). The relative, normalized ratio between phosphorylated protein and the protein itself was presented.

Anti-cancer CK2-targeted CX-4945 has been shown to inhibit the activation of CK2α and its downstream molecules, Akt and p21 [35]. As a result, the CK2-Akt signaling axis was the first among the non-Smad signals induced by TGF-β1 during EMT to be considered for testing. TGF-β1 induced the expression of CK2α protein and its nuclear translocation in A549 cells, and those inductions were strongly inhibited by the addition of CX-4945 (Figure 4A). The addition of CX-4945 also inhibited the TGF-β1-induced protein expression and phosphorylation of Akt (S129, T308 and S473) and p21. The expression of CK2β was not changed by TGF-β1 or combination with CX-4945. Additionally, even in the absence of TGF-β1, CX-4945 inhibited the activation and/or expression of molecules including CK2, Akt, p21, FAK and Src (Figure S3).

To identify TGF-β1-activated kinases, we performed a phospho-kinase array analysis; we identified that TGF-β1 strongly induced the activation of ERK in A549 cells (Figure S4). Because ERK is a non-Smad signaling molecule, we evaluated the effect of CX-4945 on the TGF-β1-induced activation of ERK. Western blot analysis confirmed that the phosphorylation of ERK1/2 was increased by TGF-β1, and that this phosphorylation was completely inhibited by 10 µM CX-4945 (Figure 4B). These results suggest that CX-4945 might inhibit both the Smad signaling pathway and non-Smad signaling pathways (e.g. Akt and ERK) during the TGF-β1-induced EMT.

### CX-4945 inhibits TGF-β1-induced activation of Wnt signaling

**Figure 5:**
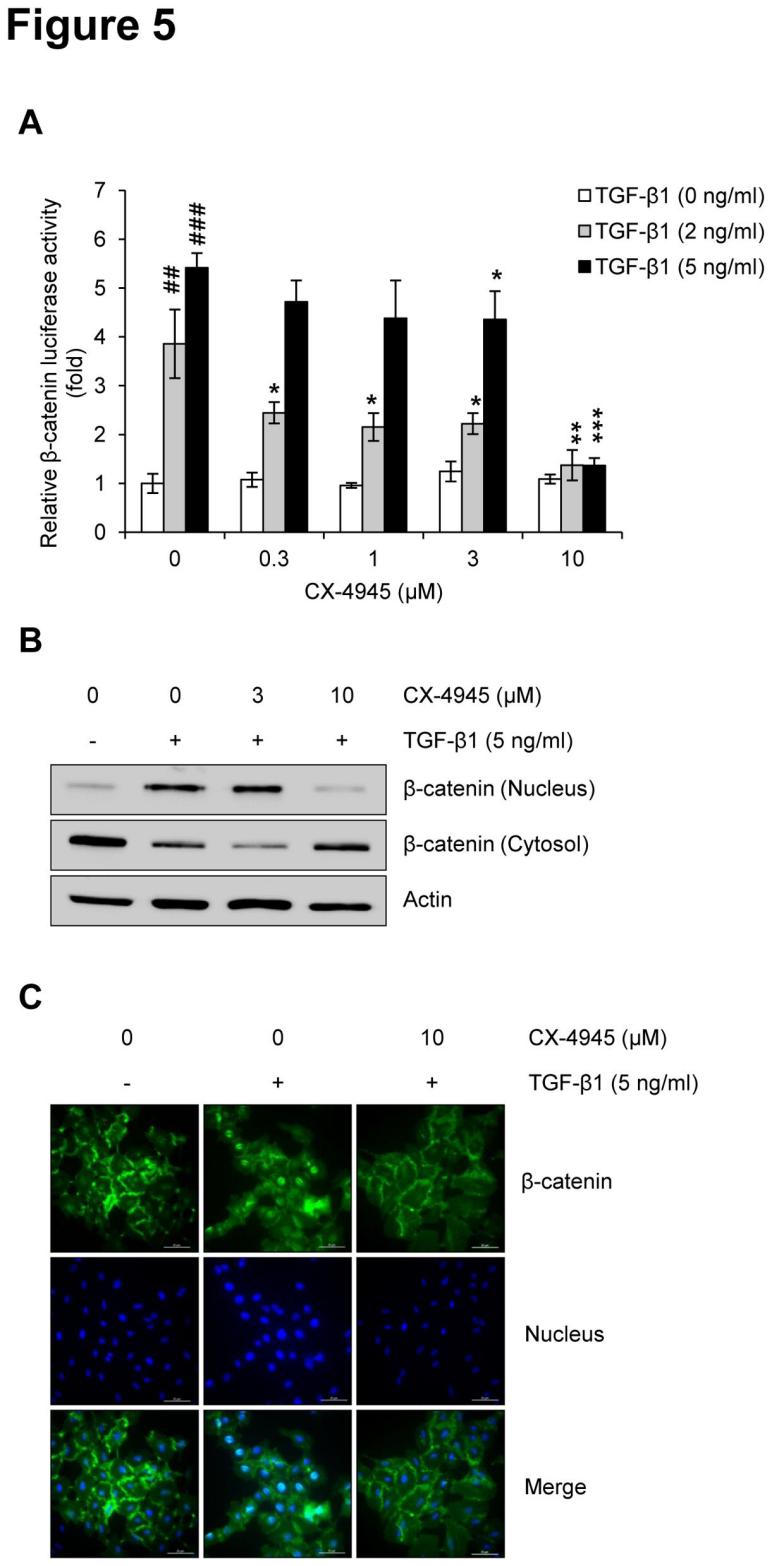
CX-4945 inhibits TGF-β1-induced Wnt signaling. The effect of CX-4945 on TGF-β1-induced Wnt activation was evaluated by measuring the transcriptional activity of β-catenin (A) and its expression in cytosolic and nuclear fractions (B). For Western blot analysis, A549 cells were treated with TGF-β1 (5 ng/ml) alone or in combination with CX-4945 in media containing 0.1% FBS for 48 h. Actin was used as a loading control. (C) Nuclear translocation of β-catenin and its inhibition by CX-4945 were confirmed by immunocytochemistry. Nuclei were counterstained with Hoechst 33342. All scale bars represent 20 µm.

During the TGF-β1-induced EMT, β-catenin (a key molecule in canonical Wnt signaling) acts as an essential component to maintain cell-cell adhesion, migration, and invasion by regulating the expression of EMT-related molecules [18]. To elucidate the involvement of Wnt signaling in the anti-EMT activity of CX-4945, we tested whether CX-4945 could inhibit the TGF-β1-induced activation of β-catenin. Results of the β-catenin luciferase assay indicated that TGF-β1 dose-dependently induced activity of β-catenin, and this induction was significantly inhibited by CX-4945 in a dose-dependent manner (Figure 5A). This finding was confirmed by Western blot analysis (Figure 5B); TGF-β1-induced nuclear translocation of β-catenin was completely inhibited by 10 µM CX-4945. These results suggest that Wnt signaling might also be involved in the inhibitory effect that CX-4945 has on the TGF-β1-induced EMT. The inhibitory effect of CX-4945 on the TGF-β1-induced nuclear translocation of β-catenin was also confirmed using immunofluorescence microscopy (Figure 5C).

## Discussion

Metastasis is a complex, consecutive, multistep process responsible for most cancer-related deaths. Cancer cells often increase their production of active TGF-β1, and its high activity is associated with highly aggressive and proliferative of cancer cells and a poor prognosis in patients. Therefore, the measurement of its serum concentration has been proposed as a complementary diagnostic test in cancer detection [36].

As a tumor promoter, TGF-β1 triggers EMT for cells to become invasive; concomitant with the loss of epithelial characteristics, cancer cells undergoing EMT acquire a mesenchymal phenotype characterized by migration and invasion [37]. Cadherin switching during the process of TGF-β1-induced EMT, the reduction of E-cadherin and the increase of N-cadherin, allows cells to become more motile and invasive [38–40]. In fact, the correlation between TGF-β1 activation and cancer invasion has been reported in several studies; transgenic expression of activated TGF-β1 converted squamous cell carcinoma into more invasive spindle cell carcinoma.

Recently, CK2α has been shown to modulate cell proliferation and invasion by regulating EMT-related genes [26]. The increased CK2α/β ratio correlates with the induction of EMT-related markers [27], suggesting that the inhibition of CK2 may be a promising therapeutic strategy to treat EMT-related disorders, including cancer metastasis. Therefore, in this study, we evaluated the effect of the CK2 inhibitor CX-4945 on the TGF-β1-induced EMT. We found that CX-4945 inhibited the TGF-β1-induced EMT characteristics, the loss of E-cadherin and the increase of N-cadherin, in A549 cells. Also, CX-4945 inhibited the TGF-β1-induced migration and invasion of A549 cells. As shown in breast cancer cells [41], TGF-β1 strongly induced the expression and activation of MMP-2 and MMP-9; those inductions were dose-dependently inhibited by CX-4945. The expression and activation of extracellular proteases such as MMP-2 and MMP-9 allow cancer cells to degrade extracellular matrix proteins, thus rendering the migration invasive [9]. Overexpression of MMPs is correlated with the progression of lung cancer [42], and the expression of either MMP-2 or MMP-9 confers a worse prognosis in early-stage lung adenocarcinoma [43]. Additionally, at the low concentration, CX-4945 significantly inhibited the TGF-β1-induced transcriptional activation of NF-κB. Since NF-κB has been well-known transcription factor to regulate the expression of genes including MMPs, this result suggested the possible involvement of NF-κB in the anti-EMT activity of CX-4945, especially its anti-invasion/migration action.

The functionally interdependent protein kinases (e.g. FAK and Src) and focal adhesion molecules (e.g. paxillin) have been involved in the TGF-β1-induced EMT [44–46]. FAK can be activated by the reduction of E-cadherin to promote the assembly of focal adhesion [47], and its upregulated activity has been reported in cancer cell lines and tissues obtained from patients with metatstic cancers [48]. The important role of FAK in cancer invasion is further supported by loss-of-function and pharmacologic inhibition studies [49,50]. As an oncogene involved in cell migration and in lung cancer tissues, paxillin was also highly expressed, amplifed, and correlated with increased EMT [51]. Additionally, breast cancer cells derived from mouse lung metastasis have exhibited an enhanced level of paxillin phosphorylation [52]. Consistent with results demonstrating the TGF-β1-mediated upregulation of FAK and paxillin, TGF-β1 in A549 cells strongly induced the expression and phosphorylation of FAK and paxillin, and CX-4945 dramatically inhibited these inductions. Although the relationships between CK2 and FAK or paxillin have not been studied in depth, here we revealed that the pharmacologic inhibition of CK2 has the potential to inhibit the TGF-β1-mediated upregulation of FAK and paxillin. The phosphorylation of paxillin can also be regulated by Akt and/or MAP kinases [53,54].

FAK and Src are extensively co-localized in the cytoplasm, and the depletion of FAK directly exhibited the inhibition of Src [55]. In this study, Src expression was not changed by TGF-β1 or TGF-β1 plus CX-4945, although CX-4945 did inhibit the TGF-β1-induced phosphorylation of Src. Src family tyrosine kinase is preferentially phosphorylated by CK2 and is activated during the TGF-β-induced EMT in mammary epithelial cells [56]. Additionally, Src-mediated regulation of E-cadherin and vimentin has been reported in several cancers [57,58]. Because there is evidence of a prominent role for Src in cancer invasion, inhibitors targeting Src are currently viewed as promising drugs for cancer therapy [59].

As well as the focal adhesion signaling pathway, TGF-β mainly activates the canonical TGF-β/Smad signaling pathway in which the Smad complex acts in the control of tumor progression, invasiveness, and metastasis during EMT by regulating the transcription of target genes in concert with other DNA-binding transcription factors such as Snail and Twist [9,60,61]. In our hands, CX-4945 dose-dependently inhibited the TGF-β1-induced phosphorylation of Smad2 and nuclear translocation of cytosolic Smad2/3. CX-4945 also inhibited the TGF-β1-mediated induction of nuclear Snail and Twist. These results suggest that TGF-β/Smad signaling is involved in the anti-EMT action of CX-4945 in A549 cells.

TGF-β stimulates the EMT through both the canonical Smad signaling pathway and a non-Smad signaling pathway [62]. The non-Smad signaling molecule Akt is regulated by CX-4945, suggesting that Akt might be an effector molecule for CK2 activity [35,63,64]. Consistent with the results reported in previous studies [26,52], TGF-β1 induced the expression and activation of both CK2α and Akt in a manner that was inhibited by CX-4945 in A549 cells. CX-4945 also inhibited the TGF-β1-mediated induction and activation of the downstream Akt effecter p21. The downregulation of p21 and p-p21 by CX-4945 has been reported in breast and pancreatic cancer cells [35].

Activation of MAP kinases also triggers the EMT in response to TGF-β1. Consistent with the results reported in a previous study [52], we also observed the TGF-β1-induced phosphorylation of ERK1/2 in A549 cells. The functional importance of ERK1/2 activation in the TGF-β1-induced EMT has been reported by several studies; its activation enhances the TGF-β1-induced EMT, accompanied by the loss of E-cadherin expression, morphological changes, and upregulation of MMPs [65–67]. Furthermore, pharmacologic inhibition of ERK1/2 resulted in a blockade of the TGF-β1-induced EMT [52,68]. In the present study, CX-4945 strongly inhibited the TGF-β1-induced phosphorylation of ERK1/2. Regulation of MMP-9 activity by Akt or ERK controls FAK-activated lung cancer metastasis [69]; furthermore, ERK-CK2-mediated phosphorylation of α-catenin promotes β-catenin transactivation and tumor cell invasion [70].

TGF-β can modulate the stability of β-catenin, and TGF-β signaling cooperates with Wnt signaling to induce EMT. Activation of Wnt signaling allows β-catenin to form a complex with TCF or LEF1, which goes on to regulate the transcription of target genes related to cell-cell adhesion and migration [18]. In the context of the EMT, Smads form a complex with LEF1 at the E-cadherin promoter, resulting in transcriptional repression [71]. Additionally, Smads and LEF1 are required for the induction of mesenchymal markers (e.g. vimentin and fibronectin). These results suggest that the mechanistic basis for the crosstalk between TGF-β/Smad and Wnt signaling might be attributable to interactions of the activated Smad complex with Wnt-activated transcription factors (e.g. TCF, LEF1, or β-catenin) [71–73]. Also, considering that the β-catenin-mediated canonical Wnt signaling pathway is upregulated by CK2 [74], CK2 has the potential to regulate TGF-β1-activated Wnt signaling. This hypothesis has been proven by the present study; TGF-β1 strongly induced the activity and nuclear translocation of β-catenin in A549 cells. However, this TGF-β1 induction was completely inhibited by 10 µM CX-4945. The expression of β-catenin, as well as c-Src and E-cadherin, is associated with vimentin activity, suggesting its transcriptional control of mesenchymal markers [57].

**Figure 6:**
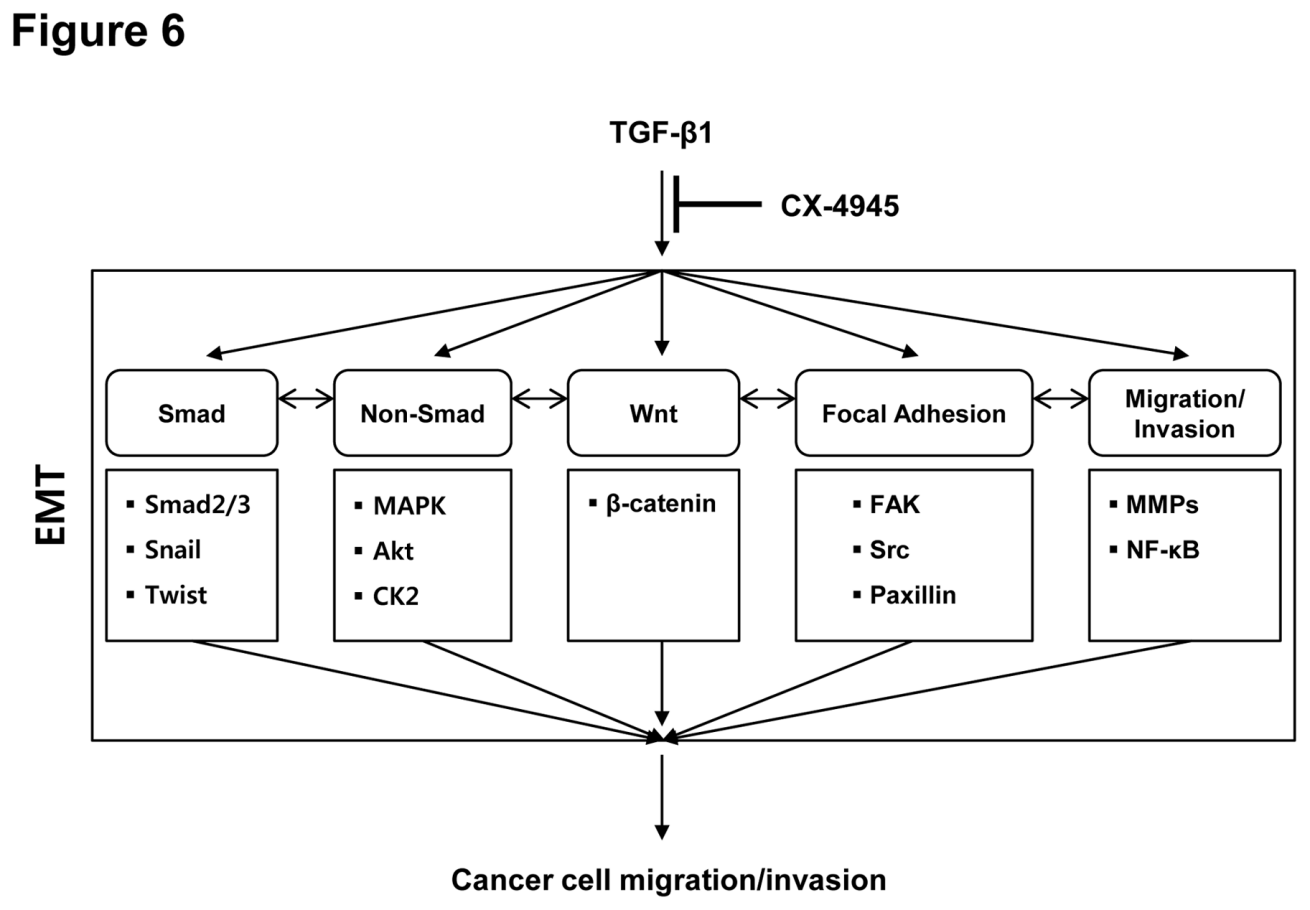
Scheme of the molecular mechanism proposed for action of CX-4945 to inhibit the TGF-β1-induced EMT. CX-4945 has the potential to inhibit the TGF-β1-induced EMT through Smad, non-Smad, Wnt, focal adhesion and MMPs/NF-κB signaling pathways.

In summary, the CK2 inhibitor CX-4945 is able to inhibit the TGF-β-activated signaling pathways that cooperatively regulate the process of EMT (Figure 6); CX-4945 inhibited the TGF-β-activated Smad, non-Smad (including Akt and ERK1/2), Wnt signaling pathway, the focal adhesion signaling pathway including FAK and Src as well as MMP and NF-κB signaling pathways. Clinical evaluation of CX-4945 in humans as a single agent to treat solid tumors and multiple myeloma has established its promising pharmacokinetic, pharmacodynamic, and safety profiles [28,35,63]. Furthermore, the second-generation CK2 inhibitor, CX-8184, has been presented within the last year. Beyond regression of tumor mass, CX-4945 may be advanced as a new therapy to treat cancer metastasis and EMT-related disorders. Its continued advancement may open the door to an entirely new class of anti-cancer treatments.

## Supporting Information

Figure S1 **CX-4945 inhibits TGF-β1-induced EMT in human normal lung fibroblasts.** CCD-18Lu (Human lung normal fibroblast) cells (1 × 10^5^ cells/ml) were seeded in a 6-well plate. After 24 h serum starvation, cells were treated with TGF-β1 (5 ng/ml) alone or in combination with CX-4945 in media containing 0.1% FBS for 48 h. Total protein (40 µg) of lysates was separated on 8~10% SDS-PAGE gel and the expression of indicated protein was measured by Western blot analysis. Actin was used as a loading control.
(TIF)

Figure S2 **Effect of CX-4945 on TGF-β1-induced transcriptional activation of NF-κB.** NF-κB reporter transfected A549 cells (5 × 10^3^ cells/well) were treated with TGF-β1 (2 or 5 ng/ml) alone or in combination with CX-4945 in media containing 0.1% FBS. After 48 h incubation, the relative luciferase activity of NF-κB reporter was evaluated by luciferase reporter assay as described in Materials and Methods. All experiments were performed in triplicate. ^###^, *p* < 0.001 (versus the control); *, *p* < 0.05; **, *p* < 0.01 (versus the cell population treated with TGF-β1 alone).
(TIF)

Figure S3 **Effect of CX-4945 on the activation and expression of signaling molecules in A549 cells.** A549 cells (1 × 10^5^ cells/ml) were treated with CX-4945 in media containing 10% FBS for 24 h. After incubation, total protein (40 µg) of lysates was separated on 8~10% SDS-PAGE gel, and the expression of indicated proteins was measured by Western blot analysis. Actin was used as a loading control.
(TIF)

Figure S4 **TGF-β1-induced phosphorylation of kinases.** (**A**) The levels of phosphorylated kinases resulting from stimulation with TGF-β1 were detected using the Proteome Profiler^TM^ Antibody Array Kit (R&D Systems, USA). Detailed experimental procedure was described in Materials and Methods section. (**B**) Reference table of human phospho-kinases as described in the array kit protocol. TGF-β1-induced phosphorylation of ERK1/2 was red-highlighted.
(TIF)
