# Supplementary figures and images for: CK2 Inhibitor CX-4945 Blocks TGF-β1-Induced Epithelial-to-Mesenchymal Transition in A549 Human Lung Adenocarcinoma Cells
Source: PLoS One. 2013 Sep 4;8(9):e74342. doi: 10.1371/journal.pone.0074342 (PMC3762800; doi:10.1371/journal.pone.0074342)

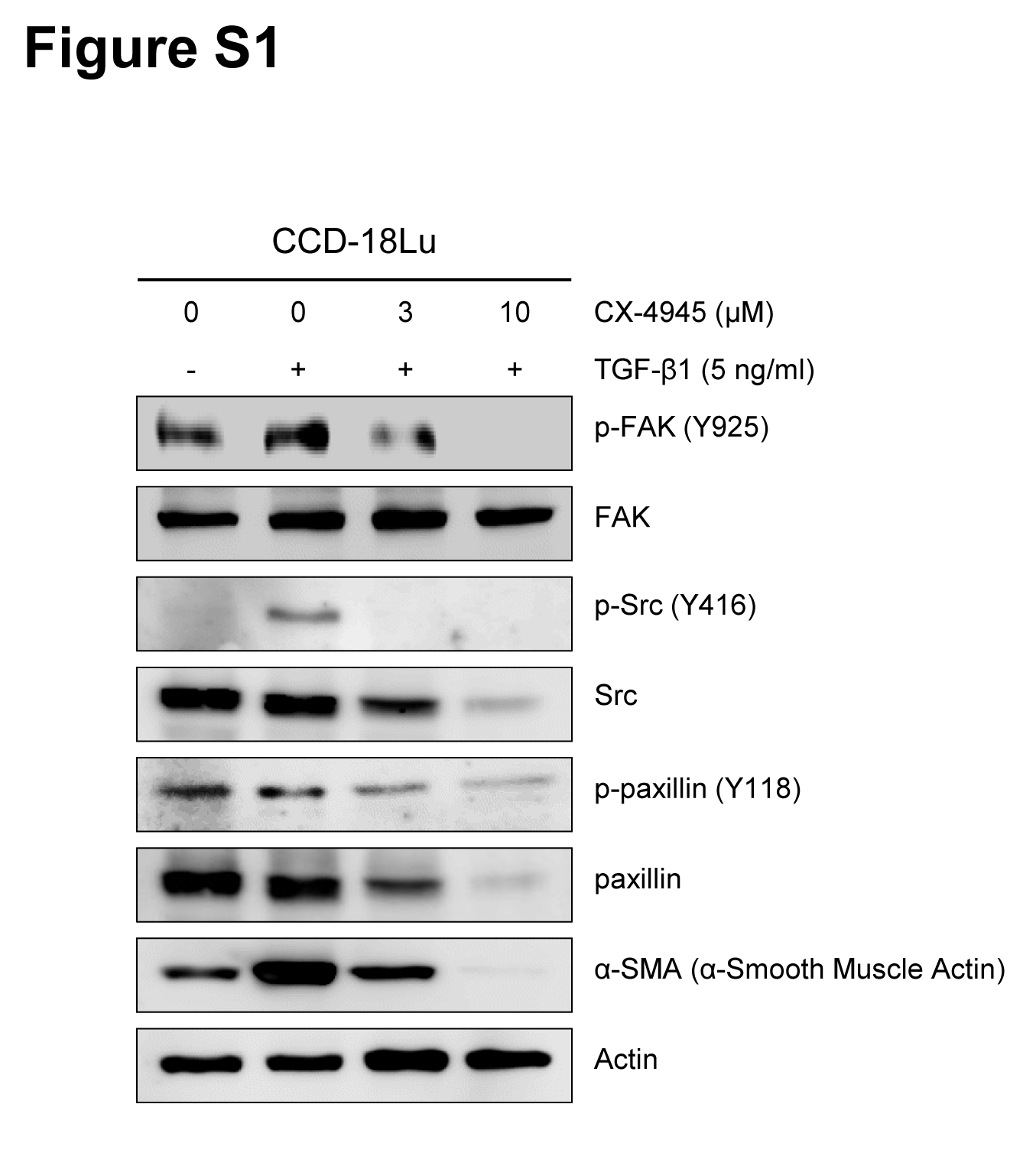

Supplement: Figure S1 — CX-4945 inhibits TGF-β1-induced EMT in human normal lung fibroblasts. CCD-18Lu (Human lung normal fibroblast) cells (1 × 10^5^ cells/ml) were seeded in a 6-well plate. After 24 h serum starvation, cells were treated with TGF-β1 (5 ng/ml) alone or in combination with CX-4945 in media containing 0.1% FBS for 48 h. Total protein (40 µg) of lysates was separated on 8~10% SDS-PAGE gel and the expression of indicated protein was measured by Western blot analysis. Actin was used as a loading control. (TIF) [file pone.0074342.s001.tif]

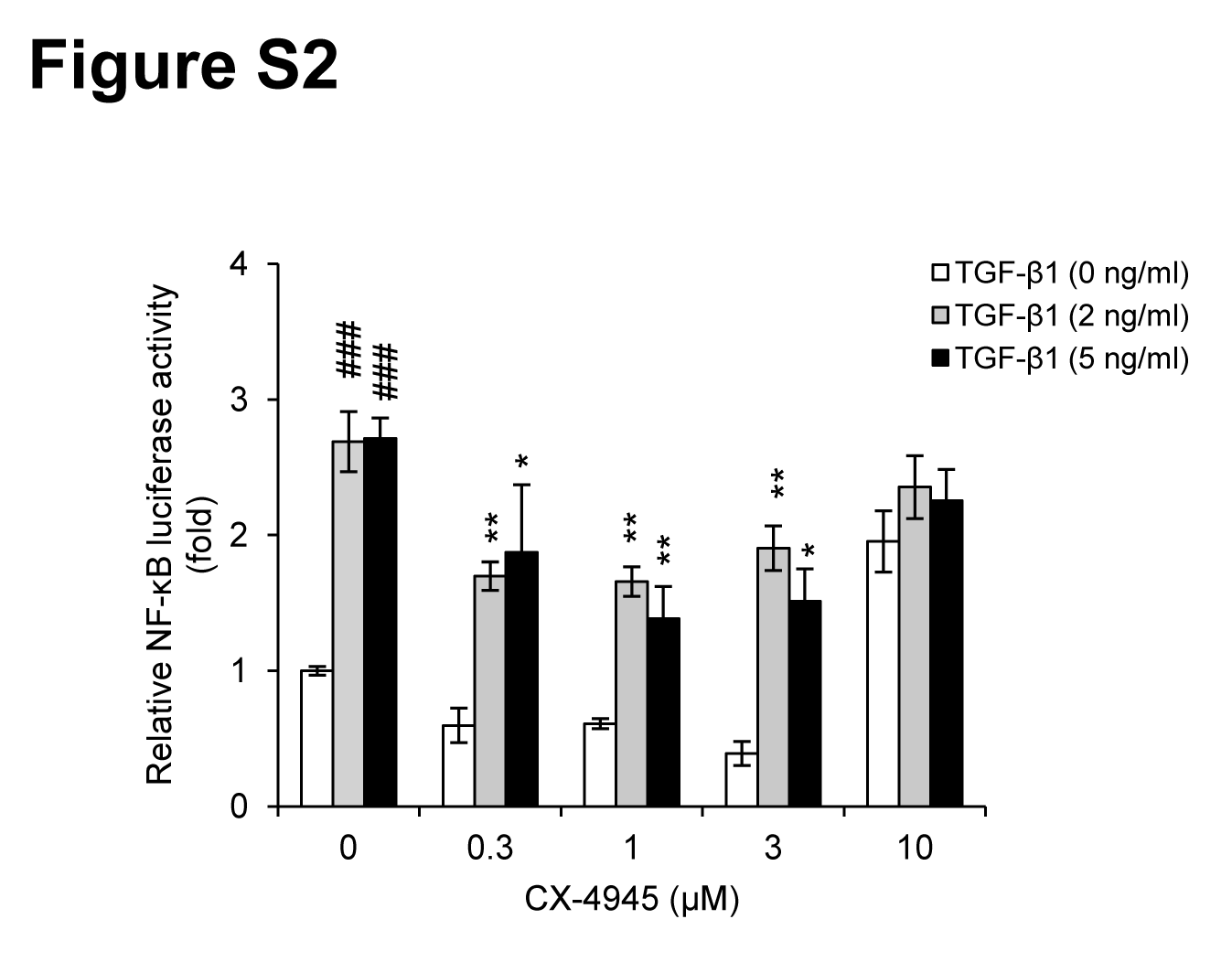

Supplement: Figure S2 — Effect of CX-4945 on TGF-β1-induced transcriptional activation of NF-κB. NF-κB reporter transfected A549 cells (5 × 10^3^ cells/well) were treated with TGF-β1 (2 or 5 ng/ml) alone or in combination with CX-4945 in media containing 0.1% FBS. After 48 h incubation, the relative luciferase activity of NF-κB reporter was evaluated by luciferase reporter assay as described in Materials and Methods. All experiments were performed in triplicate. ###, p < 0.001 (versus the control); *, p < 0.05; **, p < 0.01 (versus the cell population treated with TGF-β1 alone). (TIF) [file pone.0074342.s002.tif]

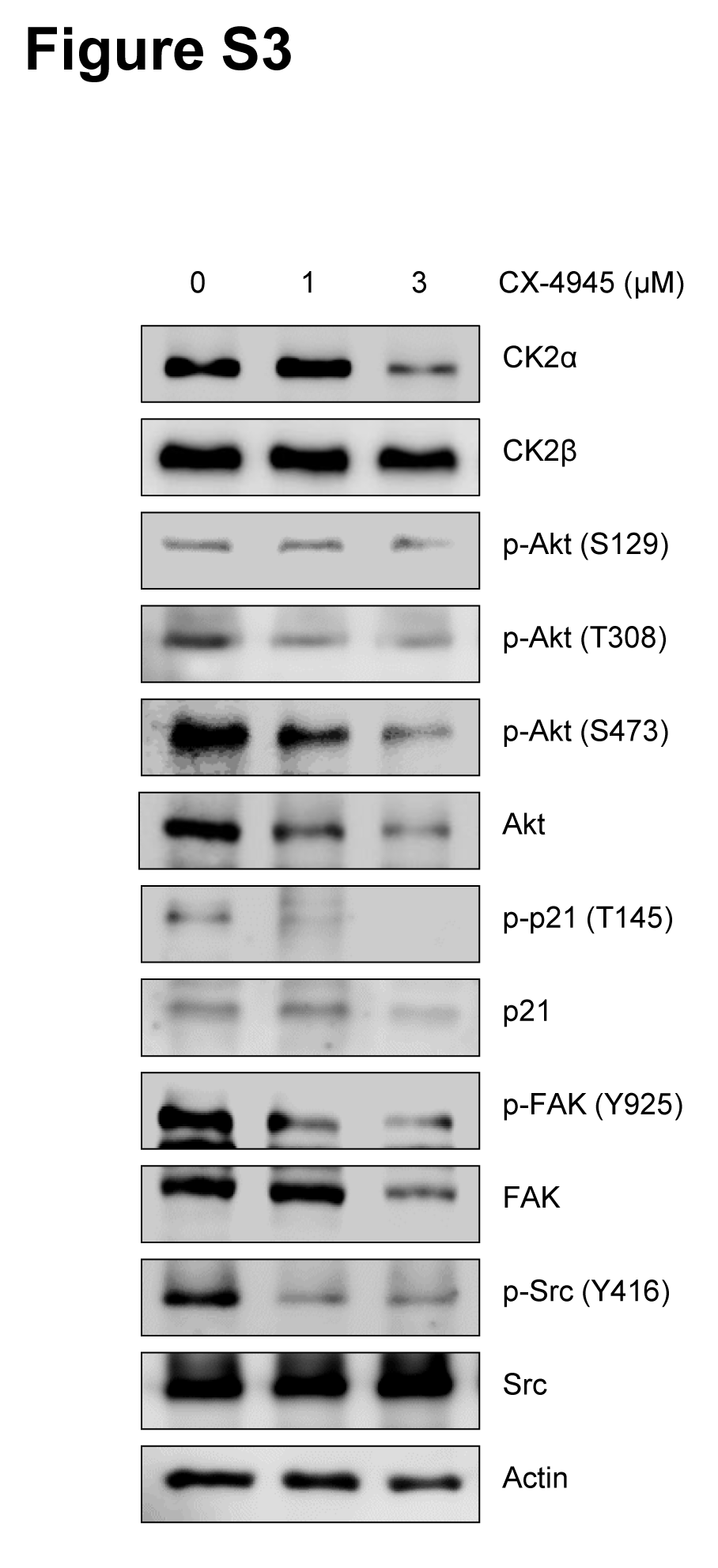

Supplement: Figure S3 — Effect of CX-4945 on the activation and expression of signaling molecules in A549 cells. A549 cells (1 × 10^5^ cells/ml) were treated with CX-4945 in media containing 10% FBS for 24 h. After incubation, total protein (40 µg) of lysates was separated on 8~10% SDS-PAGE gel, and the expression of indicated proteins was measured by Western blot analysis. Actin was used as a loading control. (TIF) [file pone.0074342.s003.tif]

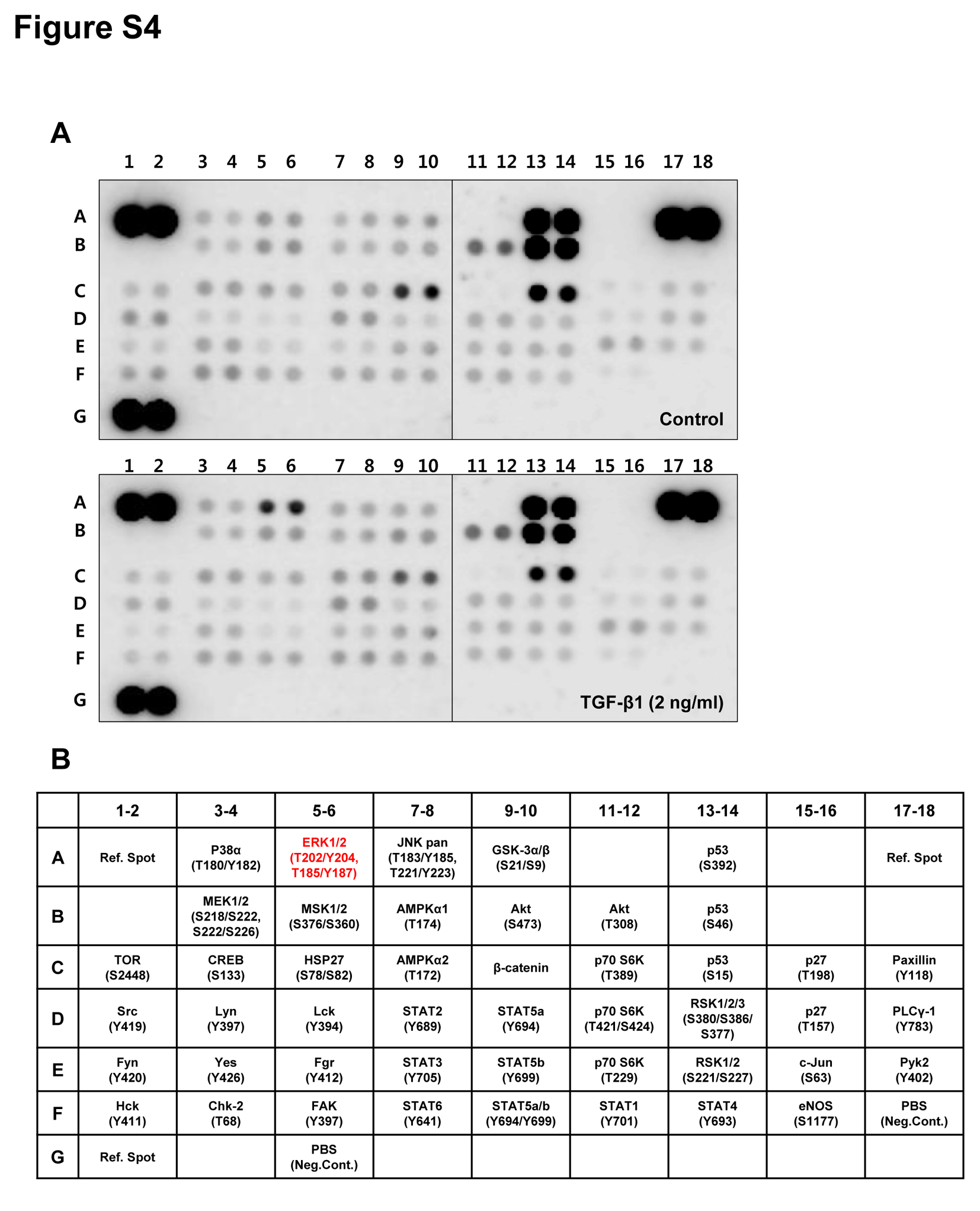

Supplement: Figure S4 — TGF-β1-induced phosphorylation of kinases. (A) The levels of phosphorylated kinases resulting from stimulation with TGF-β1 were detected using the Proteome ProfilerTM Antibody Array Kit (R&D Systems, USA). Detailed experimental procedure was described in Materials and Methods section. (B) Reference table of human phospho-kinases as described in the array kit protocol. TGF-β1-induced phosphorylation of ERK1/2 was red-highlighted. (TIF) [file pone.0074342.s004.tif]
